# Experiences and perspectives of patients with post-polio syndrome and therapists with exercise and cognitive behavioural therapy

**DOI:** 10.1186/s12883-016-0544-0

**Published:** 2016-02-10

**Authors:** Minne Bakker, Karen Schipper, Fieke S. Koopman, Frans Nollet, Tineke A. Abma

**Affiliations:** Department of Medical Humanities, EMGO+ Institute, VU Medical Center (VUmc), Amsterdam, The Netherlands; Department of Rehabilitation, Academic Medical Center Amsterdam, University of Amsterdam, Amsterdam, The Netherlands

**Keywords:** Patients, Therapists, Experiences, Qualitative study

## Abstract

**Background:**

Many persons affected with poliomyelitis develop post-polio syndrome (PPS) later in their life. Recently, the effectiveness of Exercise Therapy (ET) and Cognitive Behavioural Therapy (CBT) for PPS has been evaluated in a randomized controlled trial, but did not show a decrease in fatigue or improvement in secondary endpoints like Quality of Life and self-perceived activity limitations. The aim of this explorative study was to gain insight in the perceived effects and experiences of the interventions from the perspectives of the patients and therapists.

**Methods:**

Qualitative data were collected through semi-structured interviews with 17 patients and 7 therapists. All participants were involved in the trial. A thematic analysis of the data was performed.

**Results:**

Some patients experienced a short term enhanced endurance and a better use of energy during the day. However, in general patients did not experience a long lasting reduction of fatigue from the CBT or ET. Mainly patients of the CBT, but also some patients of the ET described an increase of self-esteem and self-acceptance. As a result, patients were sometimes better able to perform physical activities during the day. In contrast to the CBT, the ET was in general perceived by the patients as an intensive therapy, which was difficult to fit into their daily routine. Therapists of both the CBT and the ET struggled with a low intrinsic motivation of the patients in the study. This made it sometimes difficult for the therapists to follow the protocol.

**Conclusion:**

Confirming the negative quantitative study outcome, the qualitative results did not demonstrate lasting effects on fatigue. Patients did, however, experience some benefits on self-esteem and acceptance of the disease. This study showed that it is of great importance to work with feasible interventions; they should fit the patients’ needs on a practical (fit into their daily routine) and mental (fit their need for support) level.

## Background

Many people with a history of poliomyelitis report late onset neuromuscular symptoms and a decline in functional abilities decades after the acute infection. These late symptoms are referred to as post-polio syndrome (PPS) and are characterized by new or increased muscle weakness or abnormal muscle fatigability [1]. Fatigue is one of the most common complaints of PPS [2–5] and negatively influences functioning and HRQoL [6, 7]. So far, no medical treatment exists to prevent or cure PPS-related fatigue.

Recently, a single blinded randomized controlled trial (RCT) called FACTS-2-PPS was executed with the aim to reduce fatigue and to improve activities and Health Related Quality of Life (HRQoL) in people with PPS [8, 9]. Patients were randomised to either usual care (UC), exercise therapy (ET) or cognitive behavioural therapy (CBT). The aim of ET was to reduce fatigue by enhancing physical capacity. The intervention consisted of (1) a home-base aerobic training program three times a week, and (2) a supervised group-training program with muscle strengthening and functional exercises once a week over a period of four months [9]. The CBT aimed at alleviating fatigue by changing dysfunctional cognitions and behaviour related to fatigue. Dysfunctional cognitions were considered with respect to the disease, pain or fatigue, dysfunctional attention to pain and fatigue symptoms, deregulation of sleep, deregulation of physical, social and/or mental activities, and low social support and negative social interactions [9]. For each of these elements, a standardized module was available as part of the intervention. The precise details of both interventions are described elsewhere [8, 9].

The study did not demonstrate any significant benefits for decrease in fatigue and secondary endpoints like HRQoL or self-perceived activity limitations.

To gain more insight in the strong and weak points of both interventions, a qualitative study ran parallel to the trial since qualitative studies can complement the quantitative outcomes of treatment comparisons [10]. The objective of this study was to understand the perceived effects and experiences of the CBT and ET in the FACTS-2-PPS trial, from the perspective of patients as well as therapists in addition to the quantitative study outcomes.

## Methods

### Design

The present study took a descriptive qualitative research approach [11]. Qualitative research is characterised by open-ended research questions, instead of testing a hypothesis as is common in quantitative research [12] Patients of the trial as well as involved therapists (psychologists and physiotherapists) were included in this study.

### Recruitment and selection

Patients and therapists, allocated to one of the interventions, were asked by an independent investigator (who performed the randomization) to participate in the qualitative study. The patients were randomly asked to participate in the qualitative study, in order to prevent a selection bias. Still, the risk of a selection bias could not entirely be prevented, as some patients refused to participate in the interview, mainly due to lack of time. However, as the group of patients varied in age, sex and perceived fatigue, we feel confident that no selection bias occurred. Participants that agreed to an interview were then approached for an appointment by our research team. Patients were recruited until saturation of information was reached.

### Data collection

The process of data collection and analysis was iterative, meaning that the researchers started data analysis after the first interviews [13]. In this way, the emerging themes could be further explored and validated in the following interviews and in a focus group. Besides, the iterative process made it possible to determine whether saturation was reached when no new codes emerged from the data analysis.

Each patient took part in a semi-structured face-to-face interview lasting approximately 60–90 min. The interviews were conducted by two principle investigators (MB and KS). To structure the interviews and maintain conformity in the different interviews, an interview guide was used by both investigators. The interview guide consisted of semi-structured open-ended questions and more general topics and specified to the participant group (see Appendix). Interviews with patients took place at their homes, for privacy and comfort. The interviews with therapists took place at their workplace. All interviews were audiotaped and transcribed.

Patients and therapists were interviewed prior to the therapy about their expectations of the CBT or ET and shortly after the intervention period about their experiences with both interventions [14]. Interviewing prior to the intervention period was not possible in all cases as some patients (*n* = 8) started on short notice and once started, interference in the trial was for quality reasons not allowed.

An additional focus group was held to enhance the credibility of the findings, by checking the experiences mentioned in the interviews with a wider group of patients [15]. The recruitment of participants was, again, done by an independent researcher. All participants had finished the intervention and had not been interviewed about their experiences. The focus group was audiotaped and transcribed.

### Data analysis

A thematic analysis was used for this study [16]. Transcripts were separately read, re-read and coded by the two principle investigators to prevent interpretations caused by personal and professional background of the individual researcher [17]. First, the entire transcripts were read and emerging themes and sub-themes were coded. New codes in the transcripts were added to the list of codes and interviews analysed previously were read again with the new code list. Codes and coded segments were compared and different codes were combined to form the themes. The themes of both researchers were compared and discussed in the research team until consensus was reached about their relevance considering the experiences with both interventions. Relevant themes were agreed upon and for each theme the most suitable quotes were selected for the final report. These quotes underpin the conclusions of the study. To validate the findings each participant (patients and therapists) received a summary of the interview or focus group, a so-called member check [8]. All participants recognized and approved the summary of their interview or focus group.

### Ethical considerations

All patients had given their written informed consent to participate in the FACTS-2-PPS study. For this qualitative study patients were approached by the researcher for an interview. Additional spoken informed consent for the interview was given by each patient and therapist. Confidentiality was maintained using restricted, secure access to the data, destruction of audio tapes following transcription and de-identifying the transcripts. The study protocol of the RCT, including this qualitative study, was approved by the Medical Ethics Committee of the Academic Medical Center in Amsterdam.

## Results

### Description of participants

Seventeen patients and 7 therapists took part in an interview. Of the patients, 10 followed the ET and 7 the CBT. 10 patients were female, 7 were male. The age range of the patients was 54 to 72 years of age. All were native Dutch. Of the therapists, 4 were psychologists providing CBT and 3 physiotherapists providing ET. Nine patients were interviewed prior to the intervention and all patients were interviewed after the intervention. Five therapists were interviewed before they started the therapy and all therapists were interviewed after performing CBT or ET. In addition to the interviews, a focus group was held with three male and one female participant. The age range of the patients was 57 to 71 years. Similar themes and issues arose in the interviews and focus group. Patients who participated in the focus group recognized the experiences that emerged from the interviews. More details on sociodemographic and Polio characteristics of the participants are described in Koopman et al. [9].

In this paragraph the expectations before the start of the CBT and experiences with the CBT are addressed from the perspective of the patients and therapists respectively. Then the findings related to the ET are addressed from both perspectives. Representative quotes have been compiled in Tables 1 and 2. Information about the sort of interview is added, to indicate whether the interview was conducted prior (p) or after (a) the therapy.

**Table 1:** Quotes about the CBT

Quote nr	Expectations and experiences regarding the CBT	Source
	Expectations of patients	
1.	*‘There are not so many people with Post Polio, so you want to help the scientific research’*	R30 p
2.	*‘I already handle it quite well, I’m slightly satisfied, but I hope to become more balanced. I hope I’ll learn to fully accept the choices I make’*	R25 p
3.	*‘I don’t know what to expect, what can it possibly add? I don’t think you’ll improve your functioning by talking about it. I don’t have high expectations anyway…’*	R26 p
4.	‘*You could talk about it for hours, but that wouldn’t change a thing.’*	R21 a
5.	*‘[I hope] I’ll be able to accept it. That I can accept that I have this disease and that I have to keep adjusting. I find that very difficult’*	R27 p
	Experiences of patients	
6.	*‘I try to listen to my body. But I don’t feel any less tired or less pain’*	R30 a
7.	*‘I kept on going until I was so tired that I had to go to bed. But [the therapist] said “try to stay awake”. And that goes well. At the beginning it was very difficult I must say, I was exhausted. But in the end I figured out how to do it’*	R30 a
8.	*‘At a certain point I walked around the pond every day’*	R27 a
9.	*‘We started training; 20 minutes and then a short break. That was very difficult. I bought a kitchen timer, because when I start with something, I want to finish it. But with the kitchen timer it worked out quite well and it suits me well. As does the fact that I don’t sleep during the day anymore’*	R27 a
10.	*‘It felt like, and this might sound over the top, but it felt like a revelation. It taught me how to handle things. That’s really what it was’*	R27 a
11.	*‘I feel it every day, every time that I walk I think “no, I don’t have to change myself”. I always felt I had to stand up straight when I was in company, but now I thing ‘no, I don’t have to stand up straight’*	R25 a
12.	*‘It’s allright that I sometimes feel angry or sad. I used become angry when I got stuck with something, and then I felt angry because I felt angry. [The therapist] really helped me with that’*	R30 a
13.	*‘[The therapist] was a sort of mirror for me and showed me that I was no less than anyone else. I even started wearing skirts again, despite my special shoes’*	R27 a
14.	*‘Of course I’ve talked to therapists before, and every time I thought “yes, this is it!” but that feeling always faded again after three months. But this time that feeling lasts so far’*	R30 a
	Expectations of therapists	
15.	*‘I didn’t expect patients to ‘heal’ from their tiredness, because that is impossible, that is part of life. But I hoped that we would be able to teach people how to avoid the real peaks of exhaustion’*	R43 p
16.	*‘It is already an improvement for the fatigue if people realize they can do more than they expected’*	R46 p
	Experiences of therapists	
17.	*‘I had one patient who had a beautiful score at the end of the therapy. This woman with post-polio mainly worked on meaning of life questions’*	R46 a
18.	*‘[The patients] scored better than they expected. (…) One patient really improved during the therapy. He said to me at the end “I’m not sure this has improved my fatigue, but I’m very glad about that I’m doing more during the day’*	R43 a
19.	*‘That patient had a really high score on the fatigue questionnaire, but these scores are very subjective of course. She explained that that she was very tired in the evening but at the same time it was no issue at all for her that she had to lay down and watch telly’*	R42 a
20.	*‘I don’t want a treatment, I don’t need a treatment. If I would want a treatment, I would have arranged it myself. It [the fatigue] is no problem. What I can and cannot do, well too bad, but that’s just the way it is. I don’t worry about it and I figure out a way to handle it myself. I don’t have a problem so why would I need a treatment.*	R52 p
21.	*‘At the beginning it was very difficult for me that patients said that the took part in the trial because they wanted to help the research. That is not a guiding question I can work with!’*	R42 a
22.	*‘I sometimes used the protocol a bit as a guide line (…). For example with the graded activity. It is not always helpful to focus too much on increasing activity, because some that would burden people too much’*	R43 a
23.	*‘Once they started, they worked really hard’*	R43 a
24.	*‘I wasn’t familiar with this population, which made me a bit insecure at the beginning: what kind of limitations do they experience? If you want to help people you really need to be able to speak the language of the illness and know the disease. I’ve learned that for other diseases over the years, but not for this one. I had my doubts about that. But that is of course always the case if you start working with a new group of patients’*	R46 p
25.	*‘To me [the fact that we did not offer a combined therapy with ET and CBT] it feels a bit like a missed opportunity. Just CBT is sometimes not enough, especially not in rehabilitation medicine. You don’t just want to tell people that they can do it, you want to show them and let them experience that. And also the other way around; when people start training, it often triggers a much bigger process, I call that the awareness’*	R43 a

**Table 2:** Quotes about the ET

Quote nr	Expectations and experiences regarding the ET	Source
	Expectations of patients	
26.	*‘That I gain insight in how to handle my body in a good way, without getting to exhausted. I try to listen to my body, I have to because of the pain, but I think I could still learn a lot’*	R17 p
27.	*‘I hope the training will result in more strength in my legs. Normally I never cycle! It does hurt in my upper legs and my legs get tired, but I’ll try’*	R19 p
	Experiences of patients	
28.	*‘I’ve learned not to cross the line over and over again. And because of that, I’m better able to do the things I want’*	R22 a
29.	*‘The pain is still there and I’m not less tired either’*	R54 a
30.	*‘I’m less harsh to myself these days. By listening to others, I experienced more self-acceptance.(…) [The other patients] told me about their wheelchairs and that made me think ‘why not?’. My wheelchair will arrive next week’*	R17 a
31.	*‘I walk more often at home. I feel less scared, because of the training I had at the rehabilitation centre’*	R24 a
32.	‘*I just couldn’t keep up. With a lót of effort I came to a certain point, but after that I just could not do it anymore, I absolutely couldn’t…’*	R55 a
33.	‘*[Because of all the activities during the day] I am just tired in the evening. And then you have to start training at nine thirty in the evening. At a certain point I was training in my pyjama’s! That was too much’*	R21 a
	Expectations of therapists	
34.	‘*Their endurance will improve due to the training, but I’m not sure whether this will positively influence the fatigue…’*	R40 p
35.	*‘I hope this will show people that they’re still able to do this, and that they feel ‘I can keep up with this’. Hopefully that will be a start for people to keep training afterwards as well’*	R39 p
	Experiences of therapists	
36.	‘*It was quite remarkable that it was possible to train the muscles. You could see people improving over the weeks, and I hadn’t expect that. I thought they would stay on the same level. Even [that patient] who had rather weak legs improved. First in the number of repetitions and later on also in the amount of weight that he lifted’*	R40 a
37.	‘*[These patients] used to have ‘fighters spirit’ and now they suddenly have to learn not to spill their energy. For this group I therefore try to focus on únderachievement [rather than overachievement]’*	R41 a
38.	*‘It was very difficult sometimes not to advice the patients on aspects that could influence their behaviour. (…) I think I did advice some patients actually. I’m so used to advising them and I want to help the patients of course, so sometimes I might have given some extra advice when I was chatting with the patient during the training’*	R43 a

### CBT

#### Patients’ expectations

Most patients participated in the trial for the purpose of science (quote 1). They had modest expectations of the CBT. Most of these patients hoped to learn to become more satisfied with the situation and their disabilities (quote 2). A substantial group was sceptical about the contribution to their physical and mental state (quote 3). Some patients had preferred to be randomized to the ET over CBT; those patients did not see how talking about their problems would improve their physical problems and fatigue (quote 4). One patient was, however, glad to be randomized into the CBT. She hoped to become better able to accept her illness (quote 5).

#### Patients’ experiences

Although most patients did not experience a decrease in fatigue after CBT (quote 6), most experienced the CBT as valuable. This was due to other physical and/or mental improvements resulting from the therapy. Physical improvements included a better use of energy during the day (quote 7), an increase in the possibility to do some physical activities for a longer period (quote 8), less sleeping during daytime and taking more rests (quote 9).

On a mental level, patients felt considerable and more improvement than expected beforehand (quote 10). Some reported they were, due to the CBT, better able to accept their disabilities (quote 11), whereas others were more resigned with their emotions (quote 12). Considering the self-esteem, patients indicated they felt more assertive, dared better to stand up for themselves and attached less importance to other peoples’ opinion. This gave them a feeling of more freedom and the possibility to be themselves (quote 13). Patients considered the effects of the CBT to be long lasting (quote 14).

#### Therapists’ expectations

Some psychologists had, beforehand, reservations about the suitability of the protocol for the CBT. The focus of the protocol was on fatigue, whereas psychologists pointed out that it would be rather difficult to reduce it. They described fatigue as a complex issue which is hard to improve (quote 15). Others, however, were familiar with the patient group and had more positive expectations about the possibility to reduce fatigue. They felt the fatigue could be reduced as patients would become more aware of their physical abilities (quote 16). Two psychologists who had not worked with people with PPS before and found it, therefore, difficult to explicate their expectations.

#### Therapists’ experiences

Overall, the therapists were moderately positive about the CBT. Most psychologist noticed improvement in some of their patients; these patients were better able to accept their disabilities (quote 17). Some patients were positively surprised by their physical abilities as a result of graded activity increase which led to a positive experience of the CBT (quote 18). However, all therapists also experienced some difficulties with the protocol and the patient group. A frequent barrier to apply CBT, was the lack of perceived distress of the patients. All psychologists highlighted that most patients did not experience specific distress from the fatigue, nor had they needs for support at the beginning of the CBT. The fact that patients had high pre-intervention scores on the CIS fatigue questionnaire did not by definition imply a feeling of distress (quote 19). Even though all patients scored 35 or higher on the CIS fatigue questionnaire, most of the patients felt no need for a therapy for their fatigue; they did not experience the fatigue as problematic (quote 20).

Motivation was stressed across psychologists as being key to the effect of the therapy. In general, the psychologists described a low intrinsic motivation of the patients for the CBT (quote 21). Despite the lack of motivation at the beginning, most patients followed the therapy until the end. Psychologists sometimes had to deviate from the protocol, for example by trying to shift the focus to other aspects of the illness experiences, by focussing less on activity increase or by offering patients a follow up appointment (quote 22). Over the course of the therapy psychologists often noticed an increase in motivation of the patients (quote 23).

Another difficulty with the intervention for some psychologists was their lack of familiarity with the diagnosis sometimes made it more difficult for the psychologists to treat the patients, as they were unsure about the physical possibilities of the patients (quote 24). However, the vast majority were treated by therapists who were familiar with the patient group.

One of the psychologists had missed the opportunity to combine the CBT and the ET; a combination of both therapies could have done more justice to both interventions (quote 25) (Table 1).

### ET

#### Patients’ expectations

As for the CBT, patients’ primary motivation to follow the ET was for the purpose of science. However, patients described some specific expectations of the ET such as learning their physical limits (quote 26) or improving their physical state (quote 27).

#### Patients’ experiences

Most patients (*n* = 7) described some physical improvement due to the ET, such as increased muscle strength, weight loss or increased endurance. Furthermore, a couple of patients (*n* = 3) highlighted the fact that they were, due to the therapy, more aware of their limitations, resulting in a better distribution of their energy (quote 28). However, these physical improvements were perceived as a short-term outcome, which often had faded shortly after finishing the ET which did not outweigh the effort of the therapy. In general the patients did not experience decrease in fatigue (quote 29).

An increase in self-esteem and self-acceptance was mentioned by some patients as improvements on a mental level. This was sometimes the result of the ET itself, and sometimes of the conversations patients had with peers in the group session. The contact with peers helped patients to better accept their disabilities (quote 30). The ET led in some cases to an increased confidence of patients about their bodies, resulting in a rise in physical activities (quote 31).

All patients described the therapy as hard. The physical effort, the fact that cycling was often experienced as boring, and the time investment were stressed by the patients as being key to the difficulty of the therapy (quote 32) The time-investment made it hard to complete the intervention and sometimes led to drop outs (quote 33).

#### Therapists’ expectations

Physiotherapists expected the ET mainly to result in an increase of endurance of the patients. They were, however not sure whether this would result in a decrease of fatigue (quote 34). Beforehand, the physiotherapists had reservations about the possibility to establish an improvement in muscle strength considering the low frequency of the muscle strengthening exercises. Some physiotherapists expected an increase in the confidence about the physical abilities of the patients (quote 35).

#### Therapists’ experiences

Increased muscle strength was noted in some patients by the physiotherapists (quote 36). Besides, therapists identified some other gains from the therapy, such as learning how to train in the correct way and a gain in self-esteem of some patients. Beside these positive aspects, the therapists also described some difficulties with the therapy. Over-achievement was stressed by the therapists as a risk for this specific group of patients (quote 37). Besides, therapists felt that a lot of experience was needed to handle the protocol right. This was considered a downside of the intervention by experienced as well as less experienced therapists. For some physiotherapists it was difficult to stick to the protocol, some gave additional advice about the use of energy during the day although this was not included in the protocol (quote 38) (Table 2).

## Discussion

### Principle findings

Confirming the quantitative results from the FACTS2PP trial [9] patients did not experience any long lasting reduction of fatigue from the CBT or ET. However, some positive effects, on physical as well as mental level, were indicated by the patients. They experienced an increased self-esteem and self-acceptance, less sleeping during day-time and better ability to distribute their energy over the day. The main downside of the ET was that it was demanding and time consuming and therefore difficult to fit into the daily routine of patients. In contrast to the ET, patients did not report negative experiences about the CBT. Therapists experienced a low intrinsic motivation of the participants of the ET as well as the CBT, possibly due to a low experienced distress; patients mainly participated for scientific reasons rather than personal needs. This made the interventions more difficult to apply and sometimes led to small adjustments in the use of the protocol. Despite the low intrinsic motivation, therapists of the ET as well as CBT felt the intervention was useful to teach patients how to exercise and how to divide their energy during the day. A resume of the mentioned advantages and disadvantages is given in Table 3.

**Table 3:** Advantages and disadvantages of the ET and CBT

	Advantages	Disadvantages
ET		
Patients	- Physical improvement (short term) - Increased self-esteem	- Difficult to fit into daily routine (time consuming) - Exhausting - Boring
Therapists	- Improvement of training skills - Possible increase of self-esteem	- Risk of over-achievement - Experienced therapists needed
CBT		
Patients	- Better distribution of energy (less sleeping during daytime) - Increased acceptance of disabilities	/
Therapists	- Increased activity in some patients - Better distribution of energy (less sleeping during daytime)	- Patients had a low intrinsic motivation for the CBT - Protocol is focused on fatigue and activity

### Comparison with other studies

#### CBT

This study was executed parallel to a larger RCT. The main findings of our study are in line with the results of the quantitative study, as both show that patients did not point out a decrease in fatigue [9]. Our study shows, however, that patients experience benefits on self-esteem and acceptance. This is remarkable, as the quantitative study showed no increase on general self-efficacy (measured with the Dutch version of the Self-Efficacy Scale, ALCOS-16) [9] or disease acceptance (measured with the acceptance subscale of the Illness Cognitions Questionnaire, ICQ [9]. Differences between the quantitative and qualitative study might be the result of the different aims of both studies. Quantitative research is designed to test hypotheses, determine whether an intervention is leading to improvements on specific determinants. Qualitative research offers insight into emotions and experiences of people, to determine what people experience, how they feel and why. Therefore, both types of research gain in principle different kinds of information, which can lead to different outcomes. Former studies also show that differences between interviews and self-reported questionnaires can occur, even when both methods address the same concepts. Fairburn et al (1994) made a comparison between an interview and a self-reported questionnaire for people with an eating disorder and found that both results differed on some core features [18]. The differences in outcome between our qualitative study compared to the quantitative study could have multiple reasons. It is possible that the used questionnaires (ALCOS and ICQ-acceptance) were not sensitive enough for this patient group, as both questionnaires are not disease specific. Especially more complex features, such as self-esteem and acceptance, show discrepancies between interviews-based measures and self-report questionnaires [18]. It is possible that patients did objectively not increase their activities, but that the patients subjectively feel more satisfied with the way they execute activities. This would not be measured in the questionnaire, but could explain the positive experience with the interventions . Another possible explanation is that the participants of the interviews focused on aspects they thought the interviewer wanted to hear (socially acceptable answers) [19].

Beside our study, few qualitative studies addressed the experiences with CBT of people with physical problems like low back pain or fibromyalgia [16, 17]. None of these studies focused on people with PPS. Most studies that paid attention to the patients’ experiences focused on mental health problems, like depression or psychosis [20, 21].

The results of our study show that most PPS patients had low expectations and did not have a need for support or intrinsic motivation for the therapy when they started the CBT. This is a well-known phenomenon in clinical studies [22]. Different determinants exist for patients to take part in a clinical study. In this RCT patients seemed to have high altruistic motivation, which could have biased the outcome of this trial [23]. It may explain the absence of improvement in some patients since intrinsic motivation is an important factor in outcome-oriented treatments such as CBT [9].

#### ET

The results of the current study show that patients experienced the ET in general as (very) intensive. However, patients did report a (short term) increase of endurance and muscle strength and some patients described an increased self-esteem after the ET. These results were not supported by the quantitative findings of the RCT [9] which showed no increase in endurance, muscle strength or daily activity. As for the CBT, such differences between quantitative measurements and qualitative experience are more common and could be the result of the low sensitivity of the tests [18] but could also be the result of socially desirable answers during the interviews or focus groups [19]. Both focus groups and individual interviews have a risk of conducting socially desirable answers. Triangulation of different research methods (focus groups, interviews, observations, questionnaires, et cetera) can reduce the risk of this bias [24]. In interviews, the risk of “groupthink” and socially accepted answers is lower than in focus groups [25].

An important finding of our study is that some patients were not able to combine their daily activities with training three times a week. This is in line with other studies that stressed the importance of the ability to incorporate the training in one’s everyday life [26–28]. If the therapy is very time demanding or it does not fit into the daily routine, patients become less motivated and sometimes abort exercising. Besides, some patients did experience the ET as boring, lacking any stimulating factors, such as enjoyment of the therapy or signs of progress, of the training. This might also explain their low satisfaction with the ET. Such ‘intrinsic motivational factors’ are important factors for program completion [26].

In general, the group training was experienced as positive; it was perceived as valuable to talk to peers. Dodd et al (2006) describe that people with MS also benefit from a group training. Individual home training requires more discipline and might therefore be more difficult for people. Although some patients did drop out, the completion of the program was rather high. This might has to do with the high motivation of patients to participate in the trial in the first place The will of patients to finish the program in the interests of science can be quite strong [26, 28].

To prevent muscular overload, feasibility of the training schemes was weekly checked by one of the therapists by reading out the heart rate monitors and checking the log books. When necessary, adjustments to the training schemes were made. However, one of the main difficulties of the physiotherapists was to avoid the participants from overachieving during the physical training. The risk of overachievement might be specifically true for people with PPS, as they are known as over-achievers in different aspects of their live [29].

### Strengths and weaknesses of the study

Some strengths and limitations can be mentioned. One of the strengths of the study was that the perspectives of the patients as well as therapists were taken into account. This resulted in an in-depth insight in the prior expectations and experiences with both interventions from different viewpoints.

A second strength was that patients were asked prospectively about their expectations of the intervention [14]. Unfortunately, not all respondents could be interviewed before the start of the intervention; 8 were asked retrospectively about their expectations. Therefore, there is a risk that a recall bias occurred, meaning that respondents have difficulty with accurately remembering their expectations or motivation [25]. We do not have reason to assume recall bias occurred in these interviews, as the respondents explained their motivation and expectation in great detail. Furthermore, interviews were continued until saturation was reached. The data collection stopped when no new codes emerged, and therefore theoretical saturation was reached [30]. Data analysis was executed by two different researchers [31]. As both researchers came to the same conclusion from the analysis, confidence about the results was heightened.

Limitations can also be mentioned. In terms of recruitment strategy, it is possible that a selection bias occurred in the patients who agreed to take part in the interviews; some patients refused to take part in an interview, mainly due to a lack of time. We therefore might have interviewed patients who experience relatively little fatigue. It is difficult to predict the effect of this bias; it could mean that the patients included were more positive about the interventions, but it could also mean that the more severely fatigued patients would profit even more from the interventions. Our study did gather positive as well as negative experiences with the interventions and the participants varied in age, sex. Besides, we interviewed the majority of the participants of the intervention. Therefore, we are confident that we did gathered a broad spectrum of experiences.

A final limitation concerns the design of the larger RCT. As mentioned by one of the psychologists, a therapy combining the CBT with ET might have been valuable for patients, as physical and psychological aspects are often closely related. This option to combine both the ET and the CBT as one intervention was considered in the design of the RCT. There were, however, practical as well as ethical reasons to waive this combined intervention; an additional intervention would require a larger sample size and there were concerns that a combined training would put too much strain on patients’ time and physical capacity.

### Implications for clinicians

This study identified some factors that can facilitate or create barriers to the participation in CBT and ET for people with PPS. With regard to the CBT, the results of this study showed the importance to take the experienced distress and need for support regarding the fatigue into account in the selection of patients. Also, motivation of patients to take part in CBT should be checked in advance, in order to align the intervention with the needs of the patient. Without motivation or a need for support, the CBT seems do have lower chance to be successful. Considering the ET, some important aspects were the possibility for patients to fit the therapy into their daily routine concerning the length of the training as well as the intensity [32]. Besides, the intensity of the training should align with the capacity of the patient in order to prevent the patient from becoming demotivated. Motivation can also be triggered by positive feedback of the therapist, by adding ‘enjoyment’ to the training or by peer contact. Besides, it is important to determine the right training capacity. This can best be done with use of the anaerobic threshold or the rating of perceived exertion [33]. Based on the results of our study, we would recommend training under supervision and/or with peers. On the other hand, training under supervision or with peers would imply an increased time investment (traveling). Balancing advantages and disadvantages of the intervention is difficult in this perspective. An online training program might be a solution where patients can have contact with a supervisor or peers, but would not need to travel elsewhere to train.

### Future research

The results of our study show that the initial motivation of patients to take part in an RCT might be relevant to take into account. In this study, the majority of the participants took part in the RCT for scientific reasons. The low of intrinsic motivation can influence the outcomes of the trial. It might therefore be useful to select patients with an intrinsic need for reducing fatigue.

Beside a selection of participants for the trial, we would also recommend to involve patients in the trial design. Other studies have shown the value of such patient participation in research [34, 35] combine qualitative as well as quantitative research methods for such a study, as qualitative research addressing the users’ perspective on motivation, expectancies and effectiveness of interventions is valuable but still rare [36]. A description of the experiences of different users (patients as well as therapists) can provide important insights of all aspects of the intervention.

## Conclusion

The results of this qualitative study are in line with the results of the quantitative study; both show that patients did not experience a decrease in fatigue. The results of this qualitative study, however, add to this knowledge by giving insight in other experienced effects of the trial, form the perception of the patients as well as therapists. This study showed that some patients of the CBT and ET did experience an increase in self-esteem and acceptance of their disease and associated disabilities, and short term enhanced endurance and better use of energy during the day. This suggests that both interventions are potentially useful for patients with PPS. Besides, the study gained insight in what aspects of the interventions were experienced as difficult to the patients and therapists. In contrast to the CBT, the ET was in general experienced as a very intensive therapy, which was difficult to fit into the daily routine for the patients and sometimes difficult to execute for the therapists. To increase the feasibility of the training, the frequency and intensity of the training should fit the possibilities of the participants. The CBT can be valuable, provided that patients have an intrinsic motivation for the therapy. This motivation was lacked by most patients in this trial, which could at least partly explain the results of the quantitative study.

## Appendix

### Topic lists

#### Topic list patients CBT and ET prior to the therapy

Opening

- Introducing ourselves
- Aim of the project
- Aim and estimated durance of the interview
- Consent about audio tape and member check

General information

- Can you tell me something about your diagnosis (what is it, since when)
- How old are you?
- What is your educational level?
- What is your family situation? (single/married/children?
- Do you get help for your disability (professionally or non-professionally)?
- Can you tell me something about your work? Do you have a job?
- Did the diagnosis affect your job? Please explain.
- What are your most important limitations or disabilities? How do they affect your life?

Information about the therapy

- How do you feel about the provided information?
- Are there things that are not clear to you about the procedure or intervention?

Reasons to take part in the trial

- Why did you decide to take part in in the trial? Please explain

Expectations

- What are your expectations about the therapy? And what do you hope will happen?
- What are your expectations considering the therapist? What do you hope for in a therapist?
- How do you feel about the fact that you were randomized into the CBT/ET group?

Former therapies

- Have you had other therapies before ? If yes, why did you stop that/those therapies?
- What were your experiences with that/those therapies? (effects, positive aspects, negative aspects)

Other therapies

- Are you currently in therapy for your PPS? If yes, what kind of therapy?
- Are there any other trainings or things that could affect the therapy of this trial?

Rounding up

- Do you have anything to add to the interview?
- Thank you very much for your time

### Topic list therapists CBT and ET before giving the therapy

Opening

- Introducing ourselves
- Aim of the project
- Aim and estimated durance of the interview
- Consent about audio tape and member check

General information

- What is your background in education and training
- Do you have experience with this training PPS patients? Please explain
- Age

Information about the trial and therapy

- Can you tell me something about the information provided about the therapy? Is it clear to you what you have to do (the content and the process)?
- Can you tell me something about the training you followed for this therapy. Was it useful? Please explain.
- Do you feel confident about starting the training? Please explain.
- What is it like to take part in an RCT as a therapist? Please explain (positive aspects and downsides)

Motivation and expectations

- Why did you decide to take part in this trial as a therapist? How do you feel about the intervention?
- What are your expectations considering the intervention (either CBT or FT)?
- What do you expect of yourself as a therapist?
- What do you expect of the patients?

Explanations about possible effects

- Can you compare this therapy with other therapies? Is it very alike or different? Please explain.
- How do you feel about the effectiveness of this therapy; do you think it will be effective? Please explain why.
- What could be possible pitfalls or downsides of the therapy?
- Do you think this therapy could be effective to this specific group of patients (PPS)?

Rounding up

- Do you have anything to add to the interview?
- Thank you very much for your time

### Topic list patients CBT and ET after finishing the therapy

Opening

- Short reflection on the first interview
- Aim and estimated durance of the interview
- Consent about audio tape and member check

General information

- What are your most important disabilities at the moment?

Reflection

Information

- How do you feel about the information given before you started the therapy?
- Were there any unexpected things?

Experiences with the therapy

- What was is like to do the CBT/ET?
- Did you experiences any differences? Please explain
- Did you finish the therapy? What was that like?
- How do you feel, in retrospective, that you were randomised into this group?

Effectiveness of the therapy

- Do you feel you have learned anything from the therapy? What? Please explain.
- Do you feel you have benefit from the training? Please explain.
- Did you experience any downsides from the therapy? Please explain.

Ideas about effectiveness

- Why do you think this therapy was (not) effective? What elements were good in your opinion?
- Why does this therapy (not) help you?
- Can you compare this therapy to any other therapies you once had? If yes; which one was more effective and why?

Contact with therapist

- Can you tell me something about your contact with the therapist?
- What were positive and negative elements?

Peer-to-peer training

- Did you train with peers? If yes; what was that like?
- Would you have preferred to train with peers or individual? Please explain.

Homework

- Was it possible to keep up with your homework? Why (not)?
- Was there anything that helped to motivate you?
- What would have motivated you to do your homework?

Future

- Do you feel you will benefit in the long term from this therapy? Please explain.
- Will you keep practicing the things you’ve learned during the therapy? Please explain.

Suggestions

- What would you change in a future intervention?
- What would you keep from this intervention for future interventions?

Rounding up

- Would you recommend this therapy to peers? Why (not)?
- Do you have anything to add to this interview?
- Thank you for your participation

### Topic list therapists CBT and ET after giving the therapy

Opening

- Short reflection on the first interview
- Aim and estimated durance of the interview
- Consent about audio tape and member check

Reflection

Expectations

- Did it live up to your expectations? Why (not)?
- What was different? Better/worse?

Information

- Can you reflect upon the information given to you before you started giving the training?
- Was the information to you sufficient? Please explain.
- Did you feel competent enough to give the training? Please explain.
- What was it like to work in this trial for you as a therapist?

Participant’s comments

- What was it like to give this therapy?
- Did you see any changes on participants? Please give examples/explanation
- Can you give examples of positive or negative experiences?

Effectiveness

- Do you feel patients benefit from this therapy? Please explain why (not).
- Did you receive comments or negative experiences of patients?
- Did you experience any pitfalls or downsides of the therapy yourself?
- Could you compare this therapy to other therapies? In what way?

Peer-to-peer training

- Did you give an individual or peer-to-peer training? What was that like?
- Do you feel this is the right form of training for this patient group?

Homework

- Do you think people trained at home? Why do you think so?
- What do you think might be difficulties for the participants?
- What could, in your opinion, have helped the participants to proceed?

Future

- Do you think the participants will benefit on the long run from this therapy? Please explain.
- Would you recommend this therapy to other patients? Why?
- Would you like to give the therapy to other patients yourself? Please explain.

Suggestions

- Do you have any suggestions for (comparable) future interventions?
- What would you change in a future intervention?
- What would you keep from this intervention for future interventions?

Own experiences

- What have you learned as a therapist from giving this intervention?
- Were there any expectations you had to reconsider for yourself or for the patients?
- What was the contact with colleagues like?
- Would you, as a therapist, take part in an RCT again? Please explain.

Rounding up

- Do you have anything to add to the interview?
- Thank you for your input.
